# Investigation of Microstructure and Physical Characteristics of Eco-Friendly Piezoelectric Composite Thin Films Based on Chitosan and Ln_2_O_3_-Doped Na_0.5_Bi_0.5_TiO_3_-BaTiO_3_ Nanoparticles

**DOI:** 10.3390/nano14211755

**Published:** 2024-10-31

**Authors:** Jacem Zidani, Moneim Zannen, Antonio Da Costa, Oumayma Mlida, Arash Jamali, Mustapha Majdoub, Mimoun El Marssi, Anthony Ferri, Abdelilah Lahmar

**Affiliations:** 1 Laboratoire de Physique de la Matière Condensée (LPMC), Université de Picardie Jules Verne, 33 rue Saint-Leu, 80039 Amiens, CEDEX 1, France; mimoun.elmarssi@u-picardie.fr; 2 Laboratory of Interfaces and Advanced Materials (LIMA), Faculty of Sciences of Monastir, University of Monastir, Bd. of the Environment, Monastir 5019, Tunisia; moneim.zannen@fsm.rnu.tn (M.Z.); mustapha.majdoub@fsm.rnu.tn (M.M.); 3 University of Artois, CNRS, UMR 8181—UCCS—Unité de Catalyse et Chimie du Solide, 62300 Lens, France; antonio.dacostafereira@univ-artois.fr (A.D.C.); oumayma_mlida@ens.univ-artois.fr (O.M.); anthony.ferri@univ-artois.fr (A.F.); 4 Plateforme de Microscopie Électronique (PME), Hub de l’Energie, Université de Picardie Jules Verne, 15 rue Baudelocque, 80039 Amiens, France; arash.jamali@u-picardie.fr

**Keywords:** eco-friendly composites, chitosan, piezoelectric, dielectric properties, photoluminescence

## Abstract

This paper investigates the synthesis and characterization of eco-friendly piezoelectric composite thin films composed of chitosan and Ln_2_O_3_-doped Na_0.5_Bi_0.5_TiO_3_-BaTiO_3_ (NBT-BT) nanoparticles. The films were fabricated using a solution-casting technique, successfully embedding the particles into the chitosan matrix, which resulted in enhanced piezoelectric properties compared to pure chitosan. Characterization methods, such as photoluminescence spectroscopy and piezo-response force microscopy (PFM) which revealed strong electromechanical responses, with notable improvements in piezoelectric performance due to the inclusion of NBT-BT nanoparticles. X-ray diffraction (XRD) analysis revealed a pure perovskite phase with the space group R3c for NBT-BT and NBT-BT-Ln particles. Scanning electron microscopy (SEM) images showed a non-uniform distribution of NBT-BT particles within the chitosan matrix. The results also suggest that the incorporation of rare earth elements further enhances the electrical and piezoelectric properties of the composites, highlighting their potential in flexible and smart device applications. Overall, these findings underscore the potential of chitosan-based composites in addressing environmental concerns while offering effective solutions for energy harvesting and biomedical applications.

## 1. Introduction

In recent years, heightened concerns over global warming and the energy crisis have intensified the need for innovative energy systems that encompass conversion, storage, and harvesting [1,2]. Energy harvesting, a pivotal solution to the energy predicament, involves absorbing energy from diverse sources and storing it, also known as power harvesting or energy saving. This approach converts mechanical, thermal, and solar energies into electrical power, eliminating the reliance on external sources [3]. The surge in interest in energy harvesting systems, featuring piezoelectric materials, is fueled by the widespread use of portable electronic devices and wireless sensors [4]. Concurrently, global industrialization and economic development have exacerbated fossil fuel scarcity and environmental pollution, particularly the issue of plastic waste [5,6]. Addressing the global energy deficit requires improved energy utilization efficiency and advancements in energy technologies. While renewable sources such as solar, wind, and biomass energy are actively explored, their efficacy in mitigating the energy shortfall remains limited by inherent discontinuity and instability [7,8].

Piezoelectric generators hold significant promise for powering portable devices and establishing autonomous electronic systems by extracting mechanical energy. The advantages of the piezoelectric mechanism, compared to other conversion techniques, stem from its scalability and high energy density across diverse size ranges. Piezoelectric materials uniquely combine mechanical and electrical effects, where mechanical deformation induces electrical polarization (direct piezoelectric effect), and electric fields produce mechanical stress (converse piezoelectric effect). These devices have considerable technological relevance, with applications in sensors, actuators, and energy harvesting systems [9], as well as in biomedicine [10]. Advancements in piezoelectric nanostructures have expanded their use in nanocomposite materials, enhancing mechanical properties [11] and electroactive surface areas for biological scaffolds [10,12].

Polymer-based composites with enhanced electrical characteristics are being explored for their applications in capacitors, static-charge dissipation, and photovoltaic devices [13,14,15]. Their mechanical flexibility and adaptability to complex configurations make them appealing for both compact electronics and larger, geometrically specialized electrical devices [16,17]. Polymers like polyvinylidene fluoride (PVDF) are commonly used in capacitors due to their high electric breakdown strength [18,19,20]. PVDF is recognized as a prominent piezoelectric polymer in biomedical applications, attributed to its high piezoelectric constant and crystalline structure [21]. Although its piezoelectric response is notable among polymers, it remains lower than that of ceramics, with typical d33 values ranging from 13 to 28 pC/N [22]. Moreover, PVDF faces challenges in terms of adhesion, stability, and the production of uniform films [21].

Most commercially available piezoelectric generators rely on lead-based materials, particularly lead zirconate titanate (PZT), which offers enhanced piezoelectric performance in various applications [23,24]. However, the environmental and health concerns associated with these materials have intensified efforts to develop lead-free alternatives that provide comparable piezoelectric properties [25].

Recently, there has been growing interest in biomaterials as sustainable alternatives for synthesizing piezoelectric materials. The degradation of PVDF can produce toxic HF, posing risks to both the environment and human health [26]. In bioelectronics, where safety and compatibility with biological systems are crucial, traditional piezoelectric materials present significant limitations. Developing biodegradable materials that undergo natural degradation by living organisms is essential, as this reduces the need for surgical removal and enhances biocompatibility [27,28,29]. The focus on creating new piezoelectric materials with optimal biodegradability aligns with the next generation of wearable and implantable bioelectronics.

Biomaterials such as amino acids, protein-based polymers (e.g., collagen), and polysaccharides (e.g., chitin) form fibrous structures that exhibit interesting piezoelectric properties. These materials degrade into simple molecules under safe biological conditions, making them suitable for bioelectronics despite their lower piezoelectric response compared to standard materials. Optimizing their orientation and polarization can significantly improve electromechanical coupling, expanding their use in biodegradable wearable devices [30,31,32]. Substantial research has been directed at exploring biomaterials derived from biopolymers like chitin and chitosan for piezoelectric applications.

**Figure 1:**
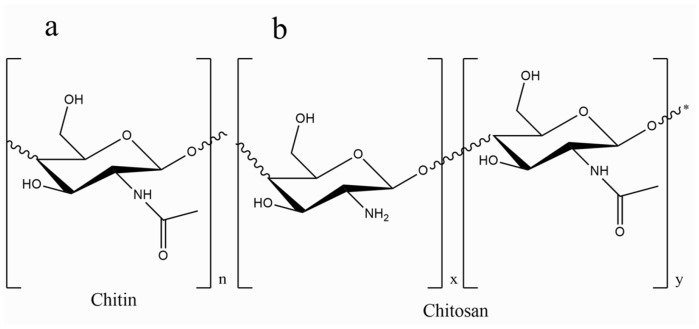
Structure of (**a**) chitin and (**b**) chitosan drawn with ChemDraw Ultra, version: 12.0.2.1076.

Chitosan (CS), derived from chitin, features a linear structure with β (1–4) linked D-glucosamine and demonstrates remarkable bioactivity and biodegradability. It is commonly found in the exoskeletons of crustaceans, fungi, and mushrooms [33,34]. The structural depiction of chitosan is illustrated in Figure 1. Approved as a food additive, chitosan has shown non-toxicity when administered orally and has proven effective in activating macrophages and attracting neutrophils [35]. Its solubility in acidic solutions and positive charge density enable interactions with a variety of anionic polymers, making it suitable for drug delivery and tissue engineering [36]. The structural similarity of chitosan to human glycosaminoglycans enhances its reactivity for biological modifications, contributing to its antibacterial and hemostatic properties, particularly in wound healing [37,38,39].

Chitosan’s mucoadhesive properties and interaction with mucous membranes have made it a key component in developing bioadhesive drug and vaccine delivery systems [40]. Its versatility in forming gels, microspheres, and nanofibers, along with its pH sensitivity and biocompatibility, positions it as a promising candidate for tissue engineering and drug delivery applications. Additionally, chitosan’s ability to form DNA complexes has driven its use in non-viral gene delivery research, emphasizing its potential in tissue engineering for skin, cartilage, and bone [41,42,43].

Integrating biopolymers like chitosan into piezoelectric systems enhances their functionality for smart devices [44,45]. Although research into chitosan’s piezoelectric applications is still developing, studies suggest that its performance rivals that of PVDF in some contexts [34,46]. This innovation aligns with sustainable development goals by offering eco-friendly alternatives to traditional energy solutions, particularly for applications in the Internet of Things (IoT) [47]. In this present investigation, we detail the fabrication of flexible nanocomposite films derived from bio-based materials by integrating Na_0.5_Bi_0.5_TiO_3_-BaTiO_3_ -lanthanides (NBT-BT-Ln) within a chitosan polymer matrix through a solution-casting method. The Na_0.5_Bi_0.5_TiO_3_-BaTiO_3_ (NBT-BT) system has garnered significant attention due to the presence of a morphotropic phase boundary (MPB) between the two phases, rhombohedral and tetragonal, particularly near x = 0.06. The introduction of BT to NBT at the MPB leads to a significant reduction in coercive fields and a noteworthy improvement in piezoelectric characteristics compared to the NBT system [48]. Recent investigations have demonstrated that the addition of rare earth (RE^3+^) elements into the 0.94NBT-0.06BT system may enhance characteristics associated with the specific rare earth element employed [49,50]. Adding Ln_2_O_3_ into BNT-BT ceramics enhances both their ferroelectric and piezoelectric properties while also heightening their fluorescence intensity, suggesting potential applications in fluorescent light-emitting devices.

## 2. Materials and Methods

The synthesis of 0.94Na_0.5_Bi_0.5_TiO_3_-0.06BaTiO_3_ powder, doped with lanthanides (Ln = Pr, Nd, and Dy), followed the conventional solid-state method using high-purity precursors: Sodium carbonate (Na_2_CO_3_, 99%), Bismuth oxide (Bi_2_O_3_, 99%), Titanium dioxide (TiO_2_, 99.9%), Barium carbonate (BaCO_3_, 99.9%), Lanthanide oxides: Praseodymium oxide (Pr_2_O_3_, 99%), Neodymium oxide (Nd_2_O_3_, 99%), and Dysprosium oxide (Dy_2_O_3_, 99%). All precursors were sourced from Alfa Aesar (Karlsruhe, Germany).

**Figure 2:**
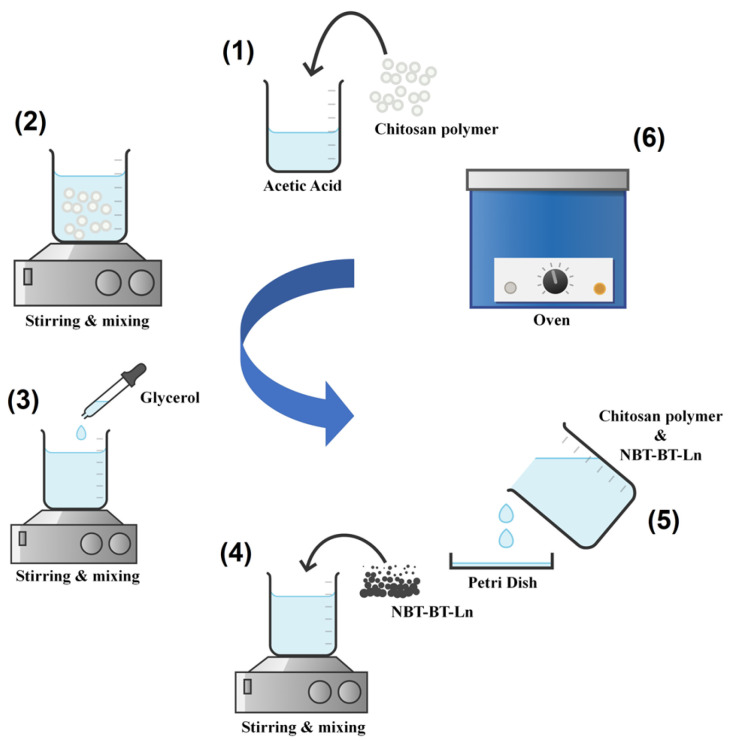
Synthesis of chitosan/NBT-BT and chitosan/NBT-BT-Ln films.

The procedure of preparing the powder, the polymer, and the nanocomposite [51,52], as well as the characterizations [53,54], are provided in the Supplementary Information. Figure 2 shows the steps of synthesis of our composite films.

## 3. Results

### 3.1. X-Ray Diffraction and Structural Analysis

**Figure 3:**
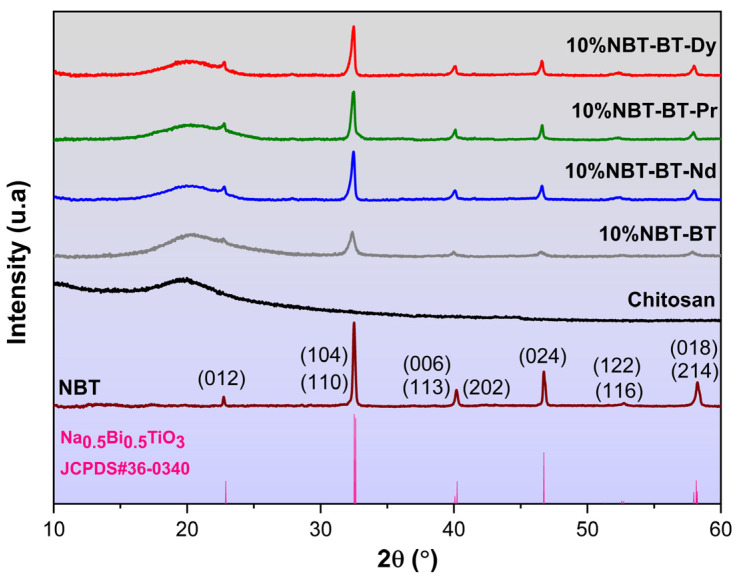
XRD of nanocomposite films chitosan, 10%/NBT-BT, and 10%/NBT-BT-Ln.

Figure 3 depicts the results of X-ray diffraction patterns for the composite membranes. All composite membranes demonstrate a singular perovskite phase associated to the ceramic particles and without any indication of secondary phase presence, as illustrated in Figure 3. The NBT-BT-Ln powder in the rhombohedral phase is classified with the space group R3c [55]. The determination of the NBT-BT-Ln crystallite size was carried out via the Debye–Scherrer formula, utilizing the Full Width at Half Maximum (FWHM) of the observed peaks, yielding a size of 19.2 nm (1) [56].

(1) D=k λβcos⁡θ

Considering the variables: *D* represents the average crystallite size, with *k* fixed at 0.9 as Scherrer’s constant; *λ* (CuKα) = 1.5406 Å indicates the wavelength of the radiation used; *β* signifies the full width at half maximum (FWHM) of the peak; and *θ* represents the Bragg angle of the most intense diffraction peak.

The distinctive CS membrane peak, evident around 2θ = 20°, is attributed to the semi-crystalline structure characteristic of chitosan [57,58]. Chitosan’s semi-crystalline characteristics arise from the dense arrangement of polymeric chains and the existence of strong intermolecular hydrogen bonds [59]. It is well-established that the structural attributes of chitosan are significantly influenced by processing treatments, including dissolving, precipitation, and drying, as well as intrinsic features such as origin, degree of deacetylation, and molecular weight. Nevertheless, the film preparation process did not notably impact the crystalline structure of chitosan, as discerned from the XRD patterns.

The additional diffraction peaks of 10%NBT-BT-Ln composite membranes become more evident, with the compositions containing Ln^3+^ situated in 22.85°, 32.52°, 40.15°, 46.69°, 52.60°, and 58.15°. These diffraction peaks were attributed to the (012), (110)(104), (113)(006)(202), (224), (122)(116), and (018)(214) planes of rhombohedral NBT-BT [60].

### 3.2. FT-IR

**Figure 4:**
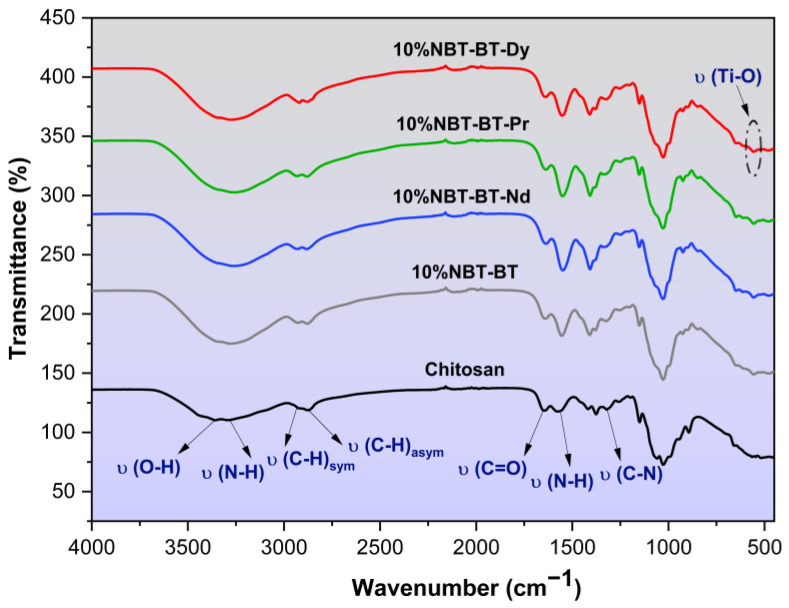
FT-IR spectra of chitosan, 10%NBT-BT, and 10%NBT-BT-Ln nanocomposites.

The infrared spectra of chitosan with a degree of deacetylation of 87.9% [34], modified with NBT-BT and NBT-BT-Ln, are illustrated in Figure 4. In this figure, the distinctive band around 559 cm^−1^ is attributed to Ti–O vibrations, characteristic of BaTiO_3_ ceramics [61]. The broadband observed at 3300 cm^−1^ in the infrared spectra of modified chitosan indicates the stretching vibrational modes of functional groups, including N–H and O–H, present in the chitosan polymer. Additionally, the bands at 2872 cm^−1^ and 2928 cm^−1^ represent the asymmetric and symmetric stretching vibrational modes of the C–H groups within the chitosan polymer chain, respectively, which are typical features of polysaccharides [62,63,64]. The presence of remaining N-acetyl functional groups is evidenced by the distinctive spectral bands at 1643 cm^−1^ (C=O stretching vibrations of amide I) and 1319 cm^−1^ (C–N stretching vibrations of amide III) [65,66]. Furthermore, a band near 1553 cm^−1^ is attributed to the N–H bending vibrations of the primary amine [67,68]. These observations align well with previous studies [69,70].

The presence of the characteristic peak of NBT-BT in all chitosan spectra suggests the formation of coordination bonds among different functional groups of chitosan and NBT-BT. This suggests that NBT-BT particles are situated between polymer chains linked through functional groups, suggesting successful incorporation of NBT-BT onto the chitosan polymer surface. FT-IR spectral bands and associated molecular vibrations are inputted in Table 1.

### 3.3. Raman Spectroscopy

**Figure 5:**
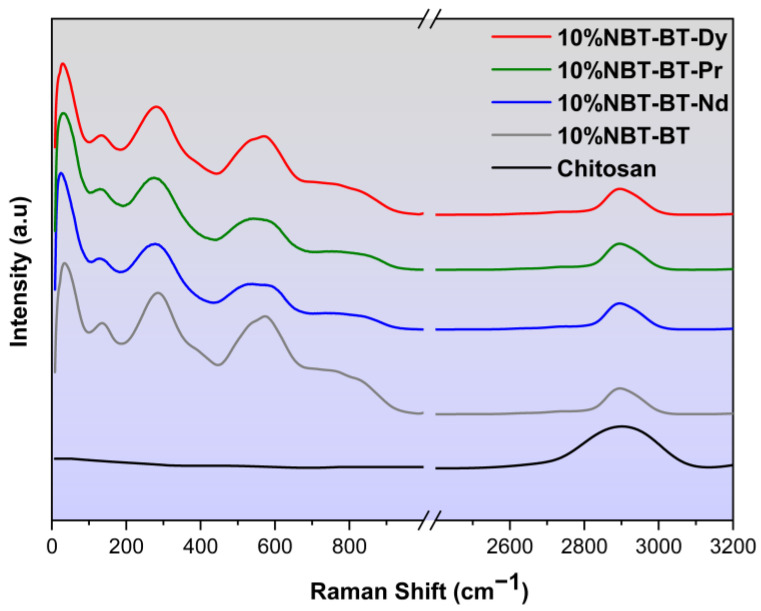
Raman of chitosan, 10%NBT-BT, and 10%NBT-BT-Ln nanocomposites.

The structure of chitosan, 10%NBT-BT, and 10%NBT-BT-Ln nanocomposites was evaluated by Raman Spectroscopy, as presented in Figure 5. The assignments obtained coincide with those reported in the literature [34,71,72]. The prominent Raman vibrations of chitosan can be assigned at 2900 cm^−1^, which were assigned to the aliphatic vibrations ν_CH_ [73,74,75].

Numerous investigations into ceramic systems [76] have studied the characteristics of the low-frequency vibrational mode to understand the influence of BaTiO_3_ doping on the NBT system; yet, despite its clear importance, several studies analyzing NBT Raman spectra have disregarded this particular frequency component [52,77].

The spectra exhibit features consistent with previously reported findings [60]. In the spectrum, different vibrational modes are observed, which aligns with observations made in other studies [78,79]. The vibrational mode at 134 cm^−1^ corresponds to the excitation of Na–O bonds at the A-site of the perovskite structure (ABO_3_). A subsequent peak at 279 cm^−1^, commonly found in various perovskites, is associated with the excitation of the TiO₆ octahedra [79]. Modes occurring within the 450 to 650 cm^−1^ range are indicative of oxygen atom displacements [76,77], while those at 765 and 842 cm^−1^ are linked to the presence of oxygen vacancies [78,79].

The cited modes, primarily involving oxygen atom displacements in perovskites while the cations remain relatively fixed, are commonly referred to [80]. All modes are wider due to increased structural disorder caused by the incorporation of Ba into NBT, with further disorder introduced by the inclusion of Lanthanide elements [81,82]. Notably, in the present study, the addition of NBT-BT and NBT-BT-Ln to the polymer CS does not lead to discernible frequency variability.

### 3.4. SEM

**Figure 6:**
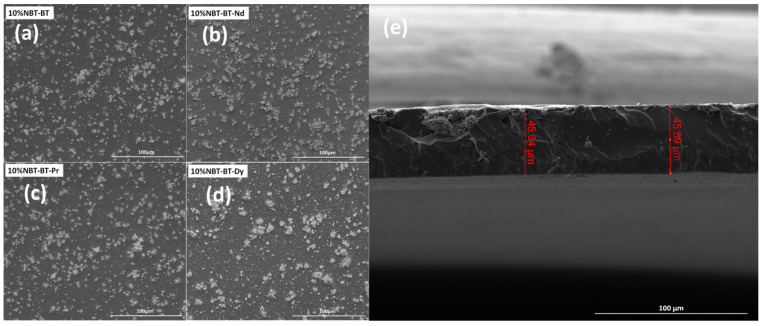
(**a**–**d**) SEM images of 10%NBT-BT and 10%NBT-BT-Ln nanocomposites and (**e**) example of cross-section of 10%NBT-BT-Dy film.

Scanning electron microscope images of the surfaces of CS/NBT-BT and CS/NBT-BT-Ln composites are depicted in Figure 6a–d. It is evident that NBT-BT and NBT-BT-Ln particles are randomly distributed and intertwined with each other, forming interconnected grains with numerous gaps in between. Although some clustering of particles can be observed on the surface, there are no obvious voids or gaps within the samples’ matrix or at the interfaces between the particles and polymer, consistent with prior literature [51,83,84]. Figure 6a–d reveals two distinct zones: one exhibiting a dark appearance and the other a light tone. The lighter particles correspond to NBT-BT-Ln particles, while the darker region represents the CS matrix. A representative cross-section of the 10%NBT-BT-Dy film is displayed in Figure 6e, revealing a uniform thickness of approximately 45 µm. The consistent thickness is confirmed by measurements taken in two different regions, demonstrating the high quality of the synthesized film. However, the surface of the film does not appear homogeneous or smooth, which is likely due to the cutting process during measurement, making it challenging to observe particles on the surface clearly.

### 3.5. Dielectric Studies

Polymers exhibit a low permittivity (low-K) characteristic. Enhancing the dielectric properties of a polymer entails incorporation of a high permittivity (high-K) ceramic substance to produce a composite. These high-K composites manifest considerable utility across diverse electronic domains, including transducers, piezo-sensors, and hydrophones [85,86]. Several literature reviews elucidating the electrical properties of various ferroelectric ceramic/polymer composites have been disseminated [87,88].

Recent attention has been directed towards high-K composite materials as prospective candidates for incorporation into high-frequency electronic systems. Emerging electronic applications like rapid computing or high-frequency telecommunication systems necessitate the integration of decoupling low-impedance power and capacitors planes directly into the packaging of integrated circuits. This necessitates the advancement of high-K materials that are compatible with circuit processing technologies [89]. The dielectric characteristics of particular synthetic polymer composites have not received much attention [90].

**Figure 7:**
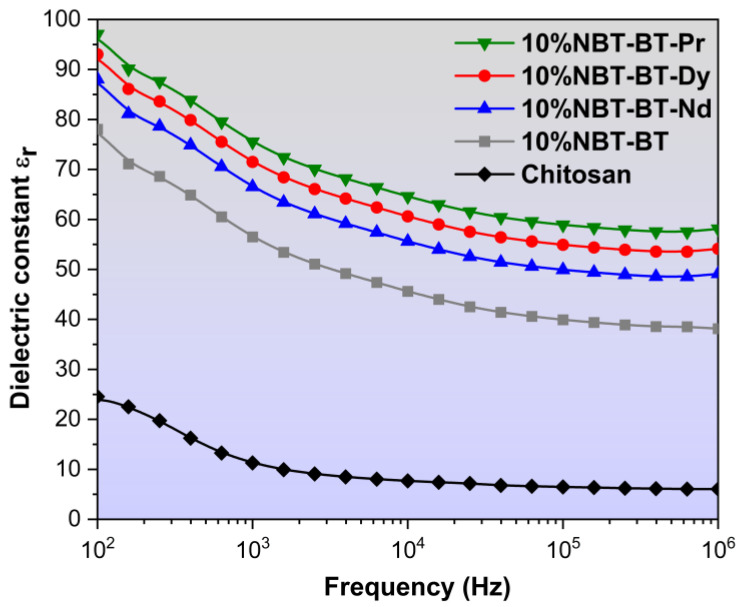
Variation of dielectric constant of chitosan, 10%NBT-BT, and 10%NBT-BT-Ln nanocomposites.

In this work, Figure 7 depicts the measured permittivity values of chitosan/NBT-BT and chitosan/NBT-BT-Ln films at ambient temperature, encompassing a frequency spectrum ranging from 10^2^ Hz to 10^6^ Hz. Each composite film exhibited an augmented dielectric permittivity compared to the pure chitosan film [34,91]. The inclusion of perovskite particles led to an enhancement in permittivity values attributed to the concomitant augmentation in interfacial space polarization within the composite materials.

However, For CS/NP, a subsequent rise across a frequency range of 100 Hz to 10^5^ Hz causes a pronounced decline in the permittivity, indicative of a relaxation phenomenon linked with the diminishing polarization stemming from the shift ranging from low-frequency to high-frequency dipolar polarization. With increasing frequency, the molecular dipoles encounter limitations in their ability to reorient swiftly enough, leading to a reduction in the permittivity [92,93]. The permittivity of the NP/CS elevates in comparison to pure CS polymer, owing to the heightened permittivity of NBT-BT [94], consequently resulting an average enhancement in electric field within the polymer nanocomposites [95].

This phenomenon can be described as follows: Upon the application of an electric field, charges accumulate at the interface between the nanoparticles (NP) and chitosan (CS) matrix. Consequently, a field is generated, polarizing the adjacent polymer matrix and giving rise to an electrical double layer around each nanoparticle. Consequently, the interfacial polarization encompassing the NP–CS hetero-interface contributes to the augmentation of the dielectric constant in the nanocomposites [96].

As depicted in Figure 7, the 10%NBT-BT-Pr composite exhibits the highest dielectric constant, approximately 100 at low frequencies, which subsequently decreases to around 60 at high frequencies. Following this, the 10%NBT-BT-Dy, 10%NBT-BT-Nd, and 10%NBT-BT composites display lower permittivity values, approximately 55, 50, and 38, respectively, at high frequencies. Additionally, chitosan exhibits a dielectric permittivity around 25 at 100 Hz, which decreases at higher frequencies, reaching a value of about 10.

Dielectric measurements show that the introduction of rare earth elements into nanocomposite films increases the dielectric constant, particularly at low frequencies, due to the presence of space charges that are highly active in these conditions. Space charges arise from processes like absorption of charged species, thermal ionization, and defect migration, with charge accumulation influenced by the interfaces between the crystalline and polymer matrix, which act as trapping sites. The addition of nanofillers enhances these interfaces, modifying the distribution of trapping sites. While all films have similar thickness and geometry, minor variations in the dielectric constant could be due to measurement conditions or differences in nanofiller distribution.

The dielectric characterization of chitosan (CS) and its nanocomposites serves as a potent tool for probing their molecular behavior, opening up possibilities for their potential applications across various fields, such as the packaging industry, fuel cells, and next-generation actuator and sensor technologies. Furthermore, by optimizing the dielectric properties of flexible films and bio-films, they could be utilized as artificial muscles in future medical treatments and play a crucial role in the development of “smart skins” for medical applications [89,97].

### 3.6. Optical Investigations

**Figure 8:**
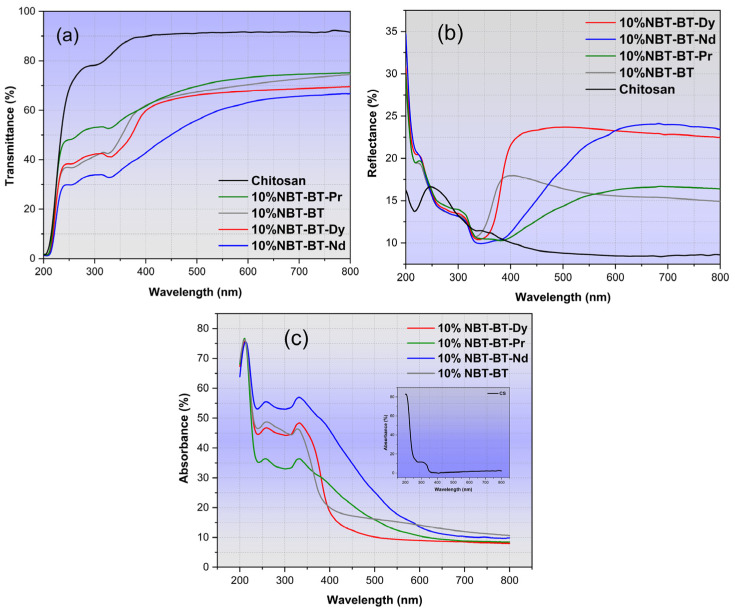
Optical response of nanocomposites: (**a**) Transmittance, (**b**) Reflectance, and (**c**) Absorbance.

Figure 8 depicts the characterization of total transmittance (T), reflectance (R), and absorbance spectrum, respectively, for various samples. These samples demonstrate the anticipated behavior typical of these composites, with a thickness approximate to 45 µm [34,98]. Notably, the composites’ spectra display a marked decrease near 350 nm, indicative of the NBT-BT band gap, as documented in the literature [99].

The transmittance of 10%NBT-BT and 10%NBT-BT-Ln films, as illustrated in Figure 8a, exhibits a substantial rise with increasing wavelength within the UV range, plateauing at a consistent maximum value in the visible spectrum. The examination of this spectrum reveals two discernible regions: (1) a transparent domain for wavelengths higher than 450 nm and (2) an absorption domain situated around 350 nm, attributed with the presence of carbonyl groups within chitosan [100,101].

Figure 8b reveals a noticeable impact of adding the NP into the CS matrix on the reflectance of the fabricated films. The reflectance of CS/NP exhibits high values in the visible range compared to the blank CS [102].

Comparatively, the transmittance of the CS/NP films decrease subsequent to the addition of NP when contrasted with the transparency of pure CS observed in previous studies [34,102]. This reduction in transparency primarily stems from light scattering by larger aggregates formed within the 10%NBT-BT films, particularly affecting shorter wavelengths.

The chitosan polymer is recognized for its capacity to absorb within the UV-visible regions, with a broad peak typically centered around 290–300 nm [100]. Figure 8c illustrates the absorbance spectrum, depicting strong UV absorption in CS/NP films, with absorption enhancement upon the incorporation of nanoparticles within the chitosan matrix [103]. The graphical representation illustrates the highest absorption occurring within the UV range, with the composite containing 10%NBT-BT-Nd exhibiting the most significant absorption in both UV and visible ranges.

**Figure 9:**
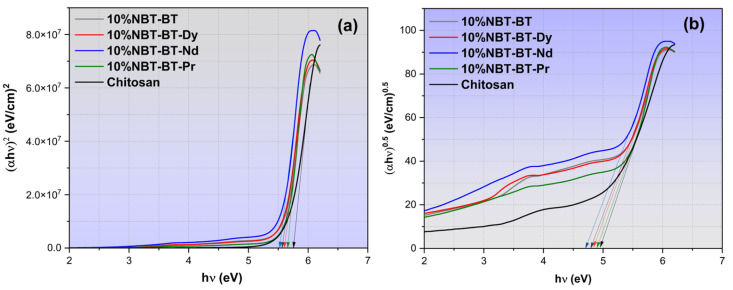
Plots of the optical bandgap illustrating (**a**) direct and (**b**) indirect status in the nanocomposites.

The increase in optical transmittance and consequent rise in absorption levels resulting from the incorporation of nanoparticles (NP) into the chitosan (CS) matrix is ascribed to heightened defect formation. This phenomenon leads to a reduction in the optical band gap of the CS/NP, as illustrated in Figure 9 [104]. Utilizing Tauc’s equation (Equation (2)), the direct and indirect optical band gaps (Eg _dir._/Eg _ind._) of both the unaltered and CS/NP films have been determined from the curves of (*αhν*)^2^ and (*αhν*)^0.5^ plotted against photon energy (*hν*), respectively, as depicted in Figure 9a,b.

(2) $${\left(\alpha h\nu \right)}^{n}=k(h\nu -Eg)$$

Here, *hν* represents the photon energy, where *h* is Plank’s constant, *α* signifies the absorption coefficient, *Eg* refers to the optical energy gap, *k* is a constant, and for direct transitions *n* = 2, while for indirect transitions *n* = 0.5.

The x-axis intercepts of the extrapolated linear segments of these curves to *hν* = 0 correspond to *Eg* values, as tabulated in Table 2. The *Eg* values obtained for direct transitions and indirect transitions of the unmodified CS film in our earlier study are 5.81 eV and 4.94 eV, respectively [34]. These values exhibited a decrease following the introduction of nanoparticles (NBT-BT and NBT-BT-Ln), consistent with previously reported findings [34,105,106]. The narrowing of *Eg* primarily arises from localized states and defects formed between both (HOMO), the highest occupied molecular orbital, and (LUMO), the lowest unoccupied molecular orbital, of the CS/NP composite due to nanoparticles doping.

**Figure 10:**
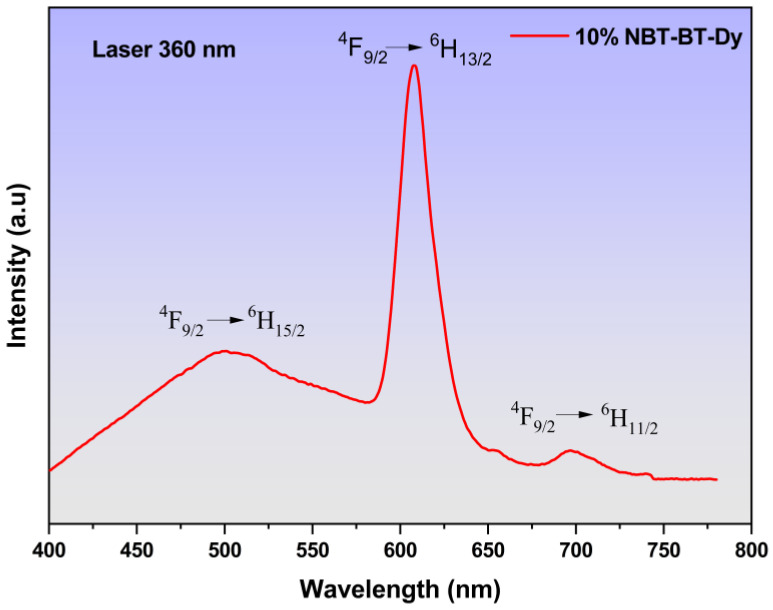
Example of emission spectra of 10%NBT-BT-Dy film.

Figure 10 illustrates the emission spectra of 10%NBT-BT-Dy at room temperature, and the 10%NBT-BT-Nd and 10%NBT-BT-Pr compounds are presented in (Figure S1), obtained through ultraviolet excitation at 360 nm. Three distinct emission bands within the visible region (VR) are discerned, with central wavelengths at 504 nm, 608 nm, and 695 nm. They correspond to transitions between 4f-4f levels in Dy^3+^ Lanthanide, specifically (^4^F_9/2_ → ^6^H_15/2_), (^4^F_9/2_ → ^6^H_13/2_), and (^4^F_9/2_ → ^6^H_11/2_), as indicated [107]. Comparative analysis with our prior research [94,108] reveals similarities in emission bands with slight wavelength shifts, potentially attributable to the polymer matrix. In numerous luminescent organic materials, including rare earth complexes, emission spectra undergo shifts based on concentration and the polymer matrix in which they are dispersed. The polymer matrix can alter the local environment and interactions surrounding rare earth ions, thereby influencing their energy levels and resultant emission wavelengths [109].

The vibrational motions of the disordered polymer host material surrounding the optically active rare earth ions can induce vibrationally-assisted electronic transitions, known as vibronic transitions. These interactions may cause shifts in the emission spectra compared to rare earth ions in a more ordered, non-polymer matrix [110]. Even though doping rare earth ions like Eu^2+^ and Dy^3+^ into a polymer matrix may not disrupt the crystal structure of the host material, the polymer environment can still affect the local coordination and interactions surrounding the rare earth ions. Consequently, this can lead to alterations in their emission properties compared to the same rare earth ions in a non-polymer matrix.

The mixture of the first emissions, blue and yellow, is recognized for producing white light in materials doped with the Dy^3+^ element, rendering it highly compelling as an activator in the fabrication of light-emitting diode (LED) systems [111]. We observed in our study the peak intensity in the yellow emission is centered at 608 nm, predominantly controlled by forced electronic dipole transitions. Notably, such transitions are permitted solely in materials characterized by polar space groups and lower symmetries [112,113]. Moreover, the strength of this emission is notably influenced by the coordination and crystal structure. Our findings reveal a solo peak at 608 nm, affirming the incorporation of Dy^3+^ ions within the A site. Conversely, the blue emission detected at 504 nm results from magnetic dipole transitions, which are comparatively less affected by the coordination environment. Leveraging these major emissions, blue and yellow, it is plausible to envision that Dy-doped CS/NBT-BT holds promise for diverse technological applications, including telecommunications, bio-labeling, medical applications, and barcode readers [114]. Additionally, its potential extends to serving in the near-infrared (NIR) regions as a solid-state laser, owing to its red emission properties.

### 3.7. Local Piezoelectric Responses

**Figure 11:**
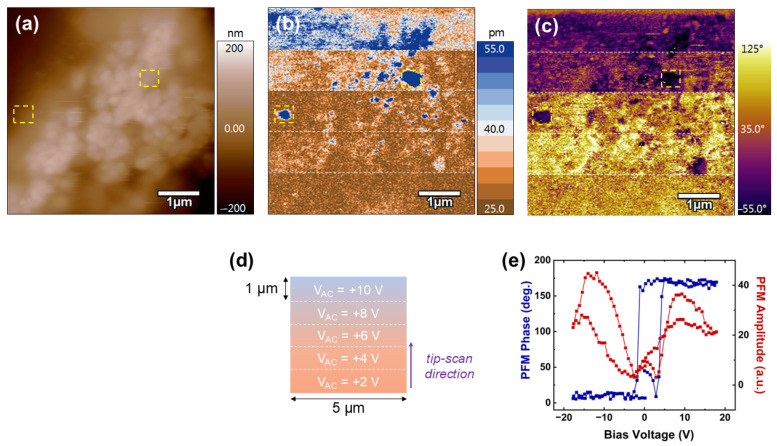
Local piezoelectric activity of the chitosan/NBT-BT composite. Images of the (**a**) AFM morphology, (**b**) PFM amplitude, and (**c**) PFM phase simultaneously recorded over the surface of the film. (**d**) Schematic representation of the PFM imaging experiments. (**e**) Representative amplitude and phase PFM loops simultaneously measured in areas marked by the yellow-dotted squares in (**a**–**c**).

**Figure 12:**
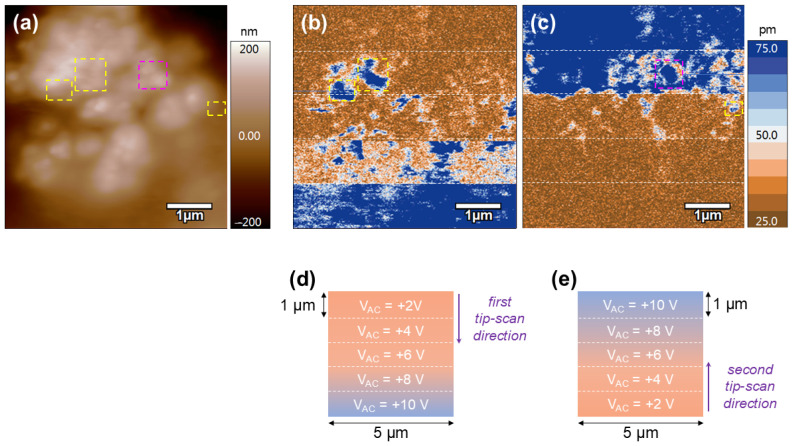
Local piezoelectric activity of the chitosan/NBT-BT-Dy composite. Images of the (**a**) AFM morphology, and (**b**,**c**) PFM amplitude recorded over the surface of the film. (**d**,**e**) Schematic representations of the PFM imaging experiments associated to the images shown in (**b**,**c**), respectively.

The morphological and electroactive properties of the chitosan/NBT-BT and chitosan/NBT-BT-Dy samples were probed at the nanoscale by the PFM technique, and the results simultaneously recorded over a 5 × 5 µm^2^ scan area are presented in Figure 11 and Figure 12, respectively. On both films, a rough surface is observed (Figure 11a and Figure 12a), in agreement with the composite polymer/ceramic nature of the studied system.

Figure 11b,c reveals the out-of-plane amplitude and out-of-plane phase of the detected PFM signal simultaneously recorded over the chitosan/NBT-BT film surface, respectively. These images represent the as-grown response of the sample under various AC electrical excitations. As schematically illustrated in Figure 11d, the PFM signal was recorded by scanning the surface from bottom to top, applying an increasing AC driving voltage from 2 V up to 10 V in 2 V increments every 1 µm. First, we observed strong PFM amplitude contrasts (blue contrasts) amid weaker responses (brown background), as highlighted by the yellow-dotted square in Figure 11b from a V_AC_ value of 6 V. The corresponding PFM phase signals are also contrasted (purple color) compared to the background of the image (yellow color). This particular behavior suggests the presence of piezoelectric perovskite particles embedded in the polymer matrix. To confirm this hypothesis, we performed spectroscopic PFM experiments, consisting of precisely positioning the AFM tip on these specific areas and then recording local piezoelectric hysteresis loops. The typical local piezo loops are shown in Figure 11e, where 180° for phase difference in phase signal and butterfly-shaped behavior for amplitude response are systematically detected, in accordance with both the switching process of the dipole moments and the piezoelectric deformation of the region probed beneath the AFM tip, respectively. In addition, an abrupt polarization reversal associated to a relatively small coercive voltage (ca. 2.7 V) is determined from the phase loops, which unambiguously evidence the nanoscale piezo-/ferroelectricity behavior of the electroactive ceramic particles. As a remark, the eventual crosstalk effect during scanning can be ruled out to explain the highly active regions in PFM images since no correlation exists between AFM morphology and piezo responses, as highlighted by the detected contrasts in yellow-dotted squares (Figure 11a–c). Now, by focusing on the background amplitude response in Figure 11b, it is noted that the deformation increases with the driving voltage, particularly from 6 V, where the contrasts gradually shift from brown to blue. Indeed, even though some highly piezo-active areas correspond to the previously identified NBT-BT particles (dark blue spots), large surrounding regions brighten and then turn blue, particularly from V_AC_ = 8 V upwards, consistent with an increase in the piezoelectric response detected by the tip, according to the associated color scale. This electrical behavior is attributed to the polymer matrix, suggesting that chitosan exhibits piezoelectric properties. This conclusion is reinforced by the fact that the matrix requires higher excitation (at least 8 V) for significant piezoelectric activity to be detected, whereas ceramic particles exhibit a piezoelectric response as early as 4–6 V. Moreover, even at voltages as high as 10 V, the chitosan matrix does not deform as much as the NBT-BT excited at 6–8 V. These results are in perfect agreement with the polymeric nature of chitosan on one hand and the perovskite nature of NBT-BT on the other, as it is well known that inorganic materials exhibit much higher piezoelectric coefficients than their organic counterparts [54]. On the other hand, the local piezoelectric behavior of the chitosan compound was already reported in the literature by using PFM analysis [115,116]. The indirect visualization of NBT-BT particles by PFM will facilitate the precise assessment of their contribution to the macroscopic performance of future composite-based electromechanical systems.

The electroactive behavior of the chitosan/NBT-BT-Ln films was also investigated at the nanoscale. The PFM results obtained over the surface of the chitosan/NBT-BT-Dy composite are presented in Figure 12. In this case, we decided to focus more on the matrix, initially performing the same kind of PFM experiment as for the chitosan/NBT-BT film, namely recording the spontaneous piezo response of the film while gradually increasing the driving voltage (from top to bottom). Then, we rescanned the same area immediately afterward under the same increasing excitation voltage (from +2 V up to +10 V), but this time from the bottom to the top of the image. These experimental conditions are schematically illustrated in Figure 12d,e, and the obtained PFM results are shown in Figure 12b,c. First, as already observed in the case of the chitosan/NBT-BT film, highly piezo-active regions (marked by yellow-dotted squares) emerge as early as 4 V of excitation, surrounded by areas with weaker response (brown regions). These regions are attributed to the NBT-BT-Dy particles, as discussed above. Moreover, specific areas with geometric shapes, suggesting the presence of particles, are observed in the PFM images, such as the one highlighted by the pink-dotted square in Figure 12c. Here, the threshold voltage required for the detection of NBT-BT is higher (approximately +8 V), which can be explained by the varying depths at which the particles are embedded in the polymer matrix Then, from the initial surface scan shown in Figure 12b, a similar behavior is observed in the matrix. Specifically, the matrix exhibits weak activity and requires a higher excitation voltage (ca. 8 V) to make the deformation under the AFM tip clearly visible (blue contrasts). When considering the results from the second scan shown in Figure 12c, we again observe a PFM signal associated with the chitosan matrix, which increases linearly as a function of the applied driving voltage. In addition, we note that the mechanical behavior of the matrix under electrical stimulus is independent of both the scanning direction and the probed region. All these results confirm the piezoelectric nature of the chitosan matrix used in this study and reveal a rather homogeneous behavior.

## 4. Conclusions

The investigation into eco-friendly piezoelectric composite thin films based on chitosan and Ln_2_O_3_-doped Na_0.5_Bi_0.5_TiO_3_-BaTiO_3_ nanoparticles yielded promising results across various aspects. The study confirmed the successful incorporation of NBT-BT nanoparticles into the chitosan matrix, as evidenced by FT-IR and RAMAN analysis. Scanning electron microscopy (MEB) revealed a random distribution of nanoparticles with a thickness around 45 µm for 10%NBT-BT-Dy as an example, enhancing interfacial interactions and contributing to improved dielectric and piezoelectric properties. Dielectric studies demonstrated that the composite films exhibited enhanced permittivity compared to pure chitosan (e.g., the 10%NBT-BT-Pr composite demonstrated a dielectric constant of approximately 100 at low frequencies), indicating the effectiveness of incorporating high-K ceramic materials to improve electrical characteristics.

The emission spectra of 10%NBT-BT-Dy composite show notable features with strong emissions in the blue and yellow regions, highlighting the impact of Dy doping on improving the optical properties. These characteristics make the composites promising for various applications, such as telecommunications, bio-labeling, medical devices, and barcode readers. Additionally, their red emission properties suggest potential use in near-infrared (NIR) solid-state lasers, broadening their scope for advanced optical technologies.

Moreover, the piezoelectric properties were analyzed using advanced techniques, showing that the chitosan matrix required higher excitation voltages (at least 8 V) to produce noticeable responses. In contrast, the NBT-BT nanoparticles exhibited piezoelectric activity at lower voltages (4–6 V). This distinction emphasizes the intrinsic characteristics of the organic and inorganic components, with the NBT-BT perovskite displaying higher piezoelectric coefficients compared to the chitosan matrix. These results offer the potential of these composites for applications in flexible electronics and energy harvesting systems. Overall, the piezoelectric capability of our chitosan/NBT-BT-Ln composites was evidenced at the nanoscale, allowing applications in electroactive devices.

## Figures and Tables

**Table 1:** FT-IR bands of nanocomposites.

Wavenumber (cm^−1^)	Band Vibration
3352	O–H stretching
3266	N–H stretching
2928	C–H symmetric
2872	C–H asymmetric stretching
1643	C=O stretching
1553	N–H bending
1319	C–N stretching
559	Ti–O stretching

**Table 2:** Direct/indirect bandgap of 10%NBT-BT and 10%NBT-BT-Ln.

Composites	*Eg _dir._* (eV)	*Eg _dir._* (eV)
10%NBT-BT-Dy	5.60	4.83
10%NBT-BT-Pr	5.65	4.89
10%NBT-BT-Nd	5.53	4.70
10%NBT-BT	5.56	4.79

## Data Availability

Data are contained within the article.

## Supplementary Materials

The supporting information can be downloaded at: https://www.mdpi.com/article/10.3390/nano14211755/s1, Materials and Methods: The procedure of preparing the powder, the polymer, the nanocomposite, and the characterizations. Figure S1: Emission spectra of 10%NBT-BT-Nd and 10%NBT-BT-Pr composites. Refs. [34,51,52,53,54] are cited in Supplementary Materials.
