# Supplementary material for: Investigation of Microstructure and Physical Characteristics of Eco-Friendly Piezoelectric Composite Thin Films Based on Chitosan and Ln2O3-Doped Na0.5Bi0.5TiO3-BaTiO3 Nanoparticles
Source: Nanomaterials (Basel). 2024 Oct 31;14(21):1755. doi: 10.3390/nano14211755 (PMC11547821; doi:10.3390/nano14211755)
Supplement: Supplementary file 1 [file nanomaterials-14-01755-s001.zip › nanomaterials-3245896-supplementary.pdf]

Investigation of Microstructure and Physical Characteristics of Eco-Friendly Piezoelectric Composite Thin Films based on Chitosan and Ln_2O_3 Doped $Na_{0.5}Bi_{0.5}TiO_3$-$BaTiO_3$ Nanoparticles.

Jacem Zidani [1, 2, *], Moneim Zannen [2], Antonio Da Costa [3], Oumayma Mlida [3], Arash Jamali [4], Mustapha Majdoub [2], Mimoun El Marssi [1], Anthony Ferri [3] and Abdelilah Lahmar [1, *]

[1] Laboratoire de Physique de la Matière Condensée (LPMC), Université de Picardie Jules Verne, 33 rue Saint-Leu, 80039 Amiens, CEDEX 1, France ; jacem.zidani@etud.u-picardie.fr (J.Z.); mimoun.elmarssi@u-picardie.fr (M.E.M.) ; abdel.ilah.lahmar@u-picardie.fr (A.L.)
[2] Laboratory of Interfaces and Advanced Materials (LIMA), Faculty of Sciences of Monastir, University of Monastir, Bd. Of the Environment, Monastir 5019, Tunisia; moneim.zannen@fsm.rnu.tn (M.Z.); mustapha.majdoub@fsm.rnu.tn (M.M.)
[3] University of Artois, CNRS, Centrale Lille, University of Lille, UMR 8181—UCCS—Unité de Catalyse et Chimie du Solide, 62300 Lens, France ; antonio.dacostafereira@univ-artois.fr (A.D.C.); oumayma_mlida@ens.univ-artois.fr (O.M.) ; anthony.ferri@univ-artois.fr (A.F.)
[4] Plateforme de Microscopie Électronique (PME) de l'Université de Picardie Jules Verne, Hub de l'Energie, 15 rue Baudelocque, 80039 Amiens, France ; arash.jamali@u-picardie.fr
* Correspondence: abdel.ilah.lahmar@u-picardie.fr; Tel.: +33-322-827-691; jacem.zidani@etud.u-picardie.fr;

Materials and Methods

Preparation of NBT-BT-Ln powder

The synthesis procedure is as follow:

- Measure the exact stoichiometric amounts of the starting reagents, mixing and grinding them in an agate mortar.
- Calcination: it is performed at 1100°C for 3 hours.
- Post-calcination Grinding: the calcined powder is ground several times to obtain a fine and uniform powder.

Preparation of Chitosan polymer

- The Chitosan was extracted from the exoskeleton of pink shrimp (P. longirostris) then cleaned with water to remove all impurities.
- Dry the shells in an oven at 100°C for 2 hours to break down the chitin structure.
- Grind the shells into a fine powder using a blender
- Transfer the chitosan powder into sealed bags and store in a freezer at 4°C for later use.

The subsequent stages in the preparation of the chitosan polymer have been detailed in a previous work [1].

Preparation of films

The composite films were prepared by incorporating NBT-BT-Ln powder into a chitosan polymer matrix via the following steps:

- Dissolving Chitosan powder in acetic acid solution.

- Adding Glycerol to the chitosan solution while stirring.
- Incorporating the prepared NBT-BT-Ln Powder to the solution
- Stir the mixture until a uniform dispersion of the particles within the matrix.
- Pour the resulting solution into a clean petri dish and place it in in an oven set to 40°C to form a thin layer.

The preparation process is similar to the methods used in previous studies [2,3].

The loading of NBT-BT-Ln based on findings from our previous work [1]. We selected the 10 wt% content of NBT-BT in this study based on findings from our previous work, where we systematically investigated the effects of varying nanofiller concentrations ranging from 2% to 10% in chitosan-NBT composites [1]. Our results indicated that the 10 wt% NBT composition provided the most favorable properties, including enhanced dielectric and optical performance, compared to lower concentrations. Therefore, we chose to focus on the 10 wt% loading in this study to build upon those optimized results and ensure the best possible performance of the composite material.

Characterizations

X-ray diffraction (XRD) analysis of the NBT-BT-Ln powder and CS/NBT-BT-Ln composite films was performed using a Bruker Discover Advance D8 diffractometer, employing CuKα radiation with a wavelength of 1.5406 Å (Karlsruhe, Germany). The composite morphology was analyzed with an Environmental Scanning Electron Microscope (SEM) using the FEI Quanta 200 FEG system from FEI Company, Hillsboro, OR, USA. Infrared spectroscopy of the composites was conducted using an IS50 ATR FTIR spectrometer, capturing the spectra in the range of 4000–400 cm^{-1}. Raman spectroscopy was performed using a Renishaw micro-Raman spectrometer LABRAM HRT 4600 HR 800 (Wotton-under-Edge, Gloucestershire, UK), with a green laser excitation source at a wavelength of 532 nm. A V-670 UV–VIS spectrophotometer was employed to analyze the optical properties of the prepared systems within the wavelength range of 200–800 nm. Dielectric properties were examined at room temperature with an impedance analyzer (TF Analyzer 2000, aixACCT, Aachen, Germany), covering a frequency range from 100 Hz to 1 MHz. Luminescence measurements were performed using photoluminescence spectroscopy (LabRAM HR Evolution), utilizing a laser with a wavelength of 360 nm. The composites surface morphology as well as their piezoelectric and ferroelectric properties were locally examined using the Atomic Force Microscopy (AFM) in Piezo-response mode (PFM). This electrical behavior was probed by means of the dual AC resonance tracking (DART) method [4] under specific environmental conditions with the aid of an MFP-3D microscope (Asylum Research/Oxford Instruments, USA). For this analysis, Pt-coated silicon tips and cantilevers with a stiffness of approximately 1.7 $N.m^{-1}$ were utilized as the conductive nano-probe (PPP-EFM, Nano-sensors). Specifically, the PFM imaging technique involved applying an AC probing voltage to the AFM tip. This allowed for the detection and analysis of spontaneous electromechanical and switching responses of the sample under investigation. Additionally, PFM in spectroscopic mode was employed, which involved recording remnant piezo-response loops (at zero bias) after applying a continuous bias signal superimposed on an

intermittent AC voltage to stimulate electromechanical response while minimizing the electrostatic contribution [5].

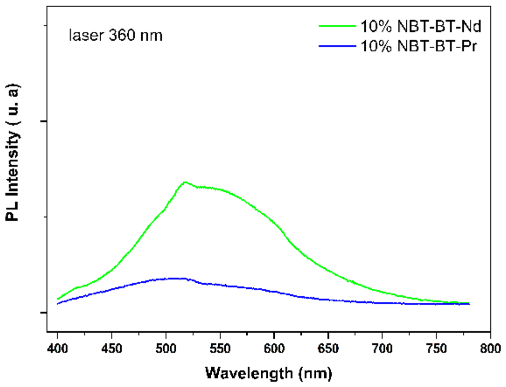

Figure S1. Emission spectra of 10%NBT-BT-Nd and 10%NBT-BT-Pr composites.

We initially aimed to study the luminescence response of all the doped samples, including those with Pr, and Nd. However, we encountered technical challenges during the measurements, which led us to successfully obtain a reliable spectrum only for the Dy-doped sample. For Pr and Nd, despite our best efforts, we struggled to detect a clear luminescence response (see figure S1). We surmise that the availability of only one type of excitation laser (360 nm) into the used device could be the cause.

References

1. J. Zidani, K. Hassine, M. Zannen, A. Zeinert, A. Da Costa, A. Ferri, J. Belhadi, M. Majdoub, M. El Marssi, A. Lahmar, Synthesis, Structural, Optical, and Electrical Characterization of Biochitosan/$Na_{0.5}Bi_{0.5}TiO_3$ Composite Thin-Film Materials, Micromachines 14 (2023) 1841. https://doi.org/10.3390/mi14101841.
2. K.D. Khalil, S.M. Riyadh, S.M. Gomha, I. Ali, Synthesis, characterization and application of copper oxide chitosan nanocomposite for green regioselective synthesis of [1,2,3]triazoles, Int. J. Biol. Macromol. 130 (2019) 928–937. https://doi.org/10.1016/j.ijbiomac.2019.03.019.
3. N.P. Maria Joseph Raj, A. Ks, G. Khandelwal, N.R. Alluri, S.-J. Kim, A lead-free ferroelectric $Bi_{0.5}Na_{0.5}TiO_3$ based flexible, lightweight nanogenerator for motion monitoring applications, Sustain. Energy Fuels 4 (2020) 5636–5644. https://doi.org/10.1039/D0SE00963F.
4. B.J. Rodriguez, C. Callahan, S.V. Kalinin, R. Proksch, Dual-frequency resonance-tracking atomic force microscopy, Nanotechnology 18 (2007) 475504. https://doi.org/10.1088/0957-4484/18/47/475504.
5. A. Ferri, S. Barrau, R. Bourez, A. Da Costa, M.-H. Chambrier, A. Marin, J. Defebvin, J.M. Lefebvre, R. Desfeux, Probing the local piezoelectric behavior in stretched barium titanate/poly(vinylidene fluoride) nanocomposites, Compos. Sci. Technol. 186 (2020) 107914. https://doi.org/10.1016/j.compscitech.2019.107914.
